# Senhance robot-assisted adrenalectomy: a case series

**DOI:** 10.3325/cmj.2022.63.197

**Published:** 2022-04

**Authors:** Nikola Knežević, Luka Penezić, Tomislav Kuliš, Toni Zekulić, Hrvoje Saić, Tvrtko Hudolin, Željko Kaštelan

**Affiliations:** 1 Department of Urology, University Hospital Center Zagreb, Zagreb, Croatia; 2 University of Zagreb School of Medicine, Zagreb, Croatia

## Abstract

We present a case series of 12 consecutive robot-assisted adrenalectomies performed from May 2019 to March 2020 by a single surgeon experienced in laparoscopy using the novel Senhance robotic system. Eleven patients had primary aldosteronism due to an adrenal adenoma, diagnosed by means of endocrinological and radiological evaluation, and 1 had a benign adrenal cyst. The robotic adrenalectomy technique is described in detail. The mean procedure time was 165.1 minutes, with robotic docking time of 11.6 minutes and console time of 98.6 minutes. The mean estimated blood loss was 47 mL, and hospital stay duration was 4.5 days. There was 1 Clavien Dindo IIIB complication and 1 patient underwent conversion to laparoscopy. All patients with adenoma had complete biochemical remission after surgery. In conclusion, the Senhance robotic system is a safe and feasible platform for benign adrenal surgery in high-volume centers.

Laparoscopic adrenalectomy is the gold standard treatment of benign adrenal tumors because of the minimally invasive approach benefits. Although robot-assisted surgery was developed to enhance these benefits, adrenalectomy has not become a widespread robot-assisted procedure, probably due to a relatively low incidence of adrenal pathology. Published reports include a small number of patients, thus limiting conclusions regarding safety and feasibility of the procedure (1). The Da Vinci Surgical System was the only robotic platform in worldwide clinical use for two decades, practically becoming a synonym for robot-assisted surgery, but nowadays novel robotic platforms are emerging. The Senhance Surgical System, introduced into clinical use in 2013, is the first new platform that has proven to be safe and feasible in various surgical, gynecological, and urological procedures (2-6). This is the first detailed case-series of 12 consecutive robot-assisted adrenalectomies performed by using the new Senhance Surgical System reporting technical and clinical aspects of the procedure.

## Case series

### Patient information and timeline

From May 2019 to March 2020, we performed Senhance robot-assisted adrenalectomy in 11 patients with primary hyperaldosteronism and one with adrenal cyst. Demographic data are shown in Table 1.

**Table 1:** Demographic characteristics of patients who underwent robot-assisted adrenalectomies performed by using the novel Senhance robotic system

Characteristic	n (%) or mean (interquartile range)
Men	6 (50)
Age (years)	48.3 (42.5-51.5)
Side	
left	7 (58.3),
right	5 (41.7)
Adenoma size (CT scan) (cm)	1.7 (1.3-2)
Incidentaloma	3 (25)
Primary hyperaldosteronism	11 (91.6)
Adrenal cyst (13 cm in diameter)	1 (8.4)

### Diagnostic assessment

The treatment plan for every patient with an adrenal tumor in our center is discussed at a meeting of the multidisciplinary tumor board, consisting of endocrinologists, urologists, oncologists, and radiologists. Patients with primary hyperaldosteronism had aldosterone-producing adenoma (APA), diagnosed with either abdominal computed tomography (CT) or magnetic resonance imaging (MRI) and saline suppression test, ambulatory salt loading test, or fludrocortisone suppression test. The diagnosis was confirmed by adrenal vein sampling. The patient with a simple non-functional adrenal cyst was diagnosed by abdominal CT.

### Therapeutic intervention

**Figure 1:**
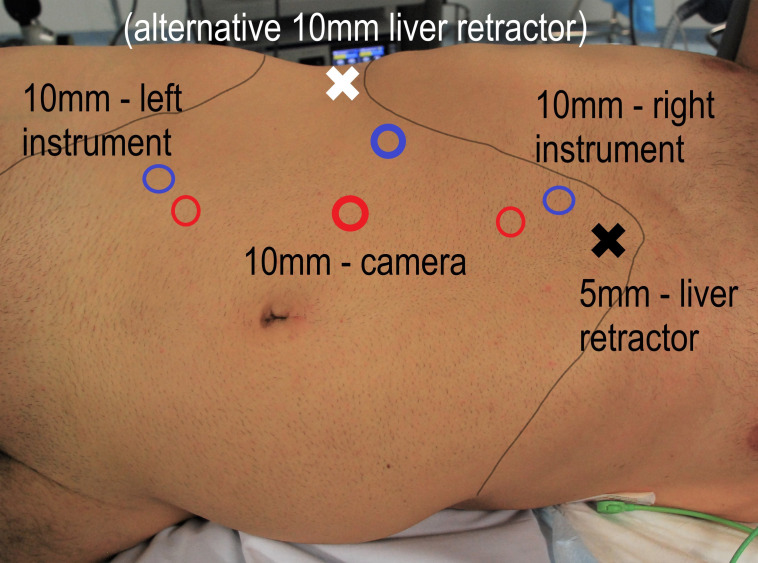
The difference between right side laparoscopic (blue circles) and robotic trocar position (red circles).

The indications and contraindications for Senhance robot-assisted adrenalectomy are the same as those for laparoscopic adrenalectomy, with the addition of body mass index >40 kg/m^2^ as a contraindication. All procedures were performed by a single high-volume surgeon with extensive laparoscopic adrenalectomy experience assisted by well-trained and experienced assistants. They all received specific training on Senhance operation and robotic system use. Our previously published 4-trocar transperitoneal laparoscopic adrenalectomy procedure (7) was modified for the robotic approach by customizing the trocar placement and adding an extra procedural step – robotic instrument docking. The laparoscopic adrenalectomy at our center is performed using a 30° optical camera, but for Senhance we used a 0° optical camera. The patient is placed in a lateral flank position. The three main trocars (camera and working trocars) are positioned in the same horizontal plane and adequately spaced – the optimal distance is a palm’s length, to avoid collision of the robotic arms outside the body. Consequently, the first 10-mm trocar is positioned in the mid-clavicular line a few centimeters caudal to the usual subcostal laparoscopic position. The second 10-mm trocar is placed in the subcostal epigastric region, and the third 10-mm trocar in the lower quadrant region (Figure 1). The trocar positions are mirrored for the left and right side adrenalectomy, except for an additional 5-mm trocar on the right side, placed in the medial line in the high epigastric region to retract the liver. The liver is retracted by using a locking grasper that holds to the lateral wall musculature. In case of a large and floppy liver that cannot be retracted with the grasper, a fourth 10-mm trocar is introduced in the mid axillary line for the fan liver retractor. The fourth assistant trocar is not necessary for the left side robot-assisted procedure. Once the optimum position is achieved, the docking of robotic instruments follows, which takes around 15 minutes at the beginning of the learning curve. With practice, the docking lasts around 7 minutes. After docking, further procedure follows the standard steps of laparoscopic adrenalectomy (7) adjusted to fit the technical requirements of the robotic system. We use three robotic arms and four robotic instruments: the monopolar L-hook, Maryland dissector, curved Metzenbaum scissors, and bipolar curved grasping forceps. The exchange of instruments is performed by the assistant and with practice takes less than 15 seconds. The adrenal gland is dissected by the operating surgeon, but the adrenal vein is clipped by the assistant. Once the adrenal gland is completely dissected and placed into a retrieval bag, the robotic instruments are removed and the bag is extracted through a lower-quadrant incision. Second-look hemostasis, if necessary, is performed laparoscopically. At the end, the drainage tube is placed, and the procedure is completed with wound closure. The drainage tube is removed once the secretion is less than 100 mL/24 h, usually on the second day. The patient is usually discharged on the fourth postoperative day.

### Follow up and outcomes

The operative and postoperative data are shown in Table 2. The procedure duration was measured from incision to skin closure, including the docking and console time. The docking time was 15 minutes for the first and 7 minutes for the last adrenalectomy in the series. The mean procedure duration was 176.6 minutes for the first three consecutive adrenalectomies and 163.3 minutes for the last three. There was 1 (8.4%) Clavien Dindo IIIB complication – bleeding on the first postoperative day, which was managed with laparoscopic revision. One patient (8.4%) underwent conversion to laparoscopy due to adhesive perinephric fat (APF). The follow-up was carried out by the leading endocrinologist. All patients had complete biochemical remission, which was confirmed by saline suppression test during follow-up.

**Table 2:** Operative and postoperative characteristics of patients who underwent robot-assisted adrenalectomies performed by using the novel Senhance robotic system

Characteristic	n (%) or mean (interquartile range)
Histology	
adenoma	11 (91.6)
cyst	1 (8.4)
Estimated blood loss (mL)	47 (8.75-62.5)
Procedure duration (min)	165.1 (146.2-188.7)
Docking time (min)	11.6 (9.5-14.3)
Console time (min)	98.6 (85-112.5)
Hospital stay duration (days)	4.5 (4-5)

## Discussion

This is the first detailed case-series of 12 consecutive robot-assisted adrenalectomies performed by using the new Senhance Surgical System reporting technical and clinical aspects of the procedure. It represents our centers’ initial experience both for the Senhance robot-assisted surgery and for robot-assisted adrenalectomy. Adrenalectomy was the first procedure to be converted to robot-assisted procedure because of our extensive laparoscopic adrenalectomy experience. Our hospital is a high-volume laparoscopic adrenal surgery center performing around 50 laparoscopic adrenalectomies per year. Patients with APA were chosen for Senhance robot-assisted adrenalectomy because of the small tumor size and simple follow-up with conventional diagnostic testing. This case series showed that the Senhance robot-assisted adrenalectomy can be performed safely and effectively within reasonable operative time.

We had only one complication, which was successfully managed with laparoscopic reoperation. It occurred in the fifth case, who underwent a right-sided adrenalectomy. The bleeding originated from a small vein in the paracaval region between the renal and adrenal vein. Such complications are rare, but common in robot-assisted adrenalectomy series (1). One similar small-sample case series (18 patients) also reported a single (5.5%) Clavien Dindo grade III complication, but it occurred in a patient with a pheochromocytoma, which was managed by conversion to open surgery and required blood transfusion (8). Our patient did not need blood transfusion, and this event did not considerably affect the length of hospital stay since the discharge was on the fifth postoperative day. We had one conversion to laparoscopy due to APF, which is a described limiting factor in robot-assisted renal surgery (9). Conversions are common, with 1%-10% conversions to laparoscopic adrenalectomy and 1% to open adrenalectomy (1).

The Senhance robotic system requires some time and experience to adapt to. In the procedures later in our series, we shortened the operative time compared with the initial procedures. If we compare the operative time of our Senhance adrenalectomy series (Table 2) with our previously published 306 laparoscopic adrenalectomy cases (median 95 ± 29 minutes [range = 45-145 minutes]) (7), two important procedure-prolonging factors can be noticed. First, this is the initial learning curve of the whole team for the Senhance Surgical System, so the optimal workflow and the position of robotic arms and assistants had to be established. Second, the procedure length was increased by modifying the operative technique, mainly the robotic instrument docking and surgeon re-scrubbing at the end of the procedure. For Senhance robot-assisted radical prostatectomy, the operative time was inversely proportional to the number of performed procedures (10,11), and for gynecological surgery operative time significantly decreased after 60-80 cases (12). Therefore, we can expect further improvements with more cases.

The Senhance Surgical System provides several benefits. First, the transition from laparoscopy to robot-assisted surgery is easy and intuitive, as has been reported by Hutchins (13). Second, the system offers several technological advancements, such as superior 3D visualization, eye tracking, haptic feedback, and comfortable ergonomics. Third, the conversion to laparoscopy can be achieved promptly and easily by simply removing the robotic arms and introducing laparoscopic instruments through the trocars. The main disadvantage of the system is the lack of space for the assistant surgeon because of the large footprint of the robotic arms. The major limitation of robotic surgery in general is the cost, not only of the initial investment, but also of the operating expenses (14). The Senhance Surgical System offers open platform strategy and reusable instruments, potentially becoming a more affordable option than the Da Vinci (15). There are only a few reports of successful Senhance robotic-assisted procedures in urology for both benign and malignant conditions (2,5,6,10,11,16-18). This report broadens the urologic catalog of Senhance robot-assisted procedures by adding adrenalectomy for benign adrenal tumors. Further prospective and randomized studies are needed to compare the Senhance robot-assisted surgery with laparoscopy, including a broader spectrum of adrenal pathology.
